# Life-Threatening Acute Chest Syndrome in a Patient With Sickle Cell Disease After Switching From Hydroxyurea Therapy to Partial Exchange Transfusions: A Case Report

**DOI:** 10.7759/cureus.20236

**Published:** 2021-12-07

**Authors:** Ann K Kvam, Henrik A Torp, Per O Iversen

**Affiliations:** 1 Department of Hematology, Oslo University Hospital, Oslo, NOR; 2 Division of Emergencies and Critical Care, Oslo University Hospital, Oslo, NOR

**Keywords:** vaso-occlusive crisis, sickle cell disease, hydroxyurea, blood transfusion, acute chest syndrome

## Abstract

Acute chest syndrome (ACS) is a severe form of vaso-occlusive crisis, which is a main feature of sickle cell disease (SCD), an inherited hemoglobinopathy. Traditionally, hydroxyurea has been the treatment of choice for SCD to prevent vaso-occlusive crises including ACS. However, hydroxyurea may be contraindicated, for example, in patients wanting to have children. We here present a young male with SCD who wanted to become a father and developed a life-threatening episode of ACS following discontinuation of hydroxyurea and switching to partial exchange blood transfusions. The patient, aged 32 years and originally from Bahrain, had been diagnosed with homozygous SCD, alpha-thalassemia, and glucose-6-phosphate dehydrogenase deficiency as a child. He had an episode of ACS with moderate severity in 2008, after which he started using hydroxyurea. From 2008 until the present, he did not experience any episodes of ACS. About six months before the present episode, he stopped using hydroxyurea and switched to partial exchange transfusions, aiming to keep hemoglobin S (HbS) below 30%. The interval between the transfusions was typically about seven to eight weeks. On the evening (day 1) before hospital admission, he developed typical symptoms and signs of vaso-occlusive crisis, and during the first day in the hospital (HbS about 55%), his pulmonary function deteriorated, and he also developed cerebral symptoms (somnolence and confusion). On suspicion of ACS, a full blood exchange transfusion was administered on day 3. He then gradually recovered clinically, and his laboratory values also normalized. He was discharged on day 10. Subsequent follow-up visits at the outpatient clinic the following month were unremarkable. Possibly, this severe episode of ACS was triggered by switching from hydroxyurea therapy to partial exchange transfusions with too long intervals between the transfusions. This novel case is a compelling reminder of the possible perils that may accompany the discontinuation of hydroxyurea, the best-documented therapy in SCD.

## Introduction

Sickle cell disease (SCD) is an autosomal recessive hemoglobinopathy mostly affecting populations residing around the equatorial belt, in particular sub-Saharan Africa and India [1]. The hallmark of SCD is chronic hemolytic anemia and episodic vaso-occlusive crisis (VOC) due to the formation of localized small emboli. A rare but serious manifestation of VOC is acute chest syndrome (ACS), which can lead to severe respiratory failure and even death [2,3].

Hydroxyurea has been the mainstay treatment for SCD for the past two decades as it protects against VOC, including ACS and death [4]. However, hydroxyurea is contraindicated in some instances, e.g., among SCD patients wanting to have children [5]. Here, we present a male SCD patient who temporarily terminated hydroxyurea, switched to regular exchange transfusions, and then developed an episode of life-threatening ACS.

## Case presentation

A 32-year-old male originally from Bahrain, and now living in Norway since 1990, was diagnosed with homozygous (HbSS) SCD in 2003 in addition to glucose 6-phosphate dehydrogenase (G6PD) deficiency. In 2007, heterozygous alpha-thalassemia (-α3.7/αα) was also detected. From the age of five years and throughout adolescence, he had several admissions to the hospital due to infections and VOC but only once with ACS (moderate form in 2008). Since he started using hydroxyurea and folic acid in 2008, he was rarely admitted to the hospital. His habitual hemoglobin (Hb) concentration has been around 9.5 g/dl. He is married, employed full-time, and became a father in 2016 after which he temporarily stopped hydroxyurea and received partial exchange transfusions usually every five weeks to keep HbS below 30%. After the birth of a healthy child, he resumed hydroxyurea.

At the end of 2020, he decided to stop hydroxyurea to become a father again. We, therefore, gave him partial exchange transfusions every seven to eight weeks according to the patient’s preference. We targeted an HbS fraction that is < 30%. He had an uneventful period until an evening at the end of July 2021 (day 1) when he suffered pain, which is located mostly in the hip region. The pain exacerbated during the night, so he was admitted to the hospital on the following morning (day 2), and the treatment was started with intravenous saline and opioids. However, his condition deteriorated with increasing pain, the onset of fever (38.5-39°C), rapid respiratory rate, reduced oxygen saturation, and worsening of laboratory parameters (Table 1). 

**Table 1:** Blood laboratory values during the course of the acute chest syndrome in our patient Ref.: Reference laboratory values at Oslo University Hospital; Hb: hemoglobin; LDH: lactate dehydrogenase; CRP: C-reactive protein; INR: international normalized ratio; aPTT: activated partial thromboplastin time; proBNP: pro-brain natriuretic peptide; PaO_2_: arterial oxygen pressure; PaCO_2_: arterial carbon dioxide pressure; N/A: data not available.

Day	Hb (g/dl)	HbS (%)	LDH (U/l)	Bilirubin mmol/l	CRP (mg/l)	Creatinine (mmol/l)	INR	aPTT (s)	Fibrinogen (g/l)	D-dimer (mg/l)	proBNP (ng/l)	PaO_2_ (kPa)	PaCO_2_ (kPa)
2	10.1	55	257	49	3	84	N/A	N/A	N/A	N/A	N/A	N/A	N/A
3	7.1	41	1351	93	99	90	N/A	37	N/A	N/A	N/A	7.9	5.8
4	12.1	8	788	65	188	93	N/A	34	4.2	11	N/A	11.5	5.4
5	10.0	N/A	760	57	112	68	1.4	32	5.7	4.3	338	N/A	N/A
10	9.3	N/A	340	16	50	70	N/A	N/A	N/A	N/A	189	N/A	N/A
33	9.9	N/A	198	61	5.2	70	N/A	N/A	N/A	N/A	N/A	N/A	N/A
Ref.	13.4-17.0	0	105-205	5-25	<4	60-105	0.9-1.2	22-30	1.9-4.0	<0.50	<85	10.0-14.0	4.7-6.0

**Figure 1:**
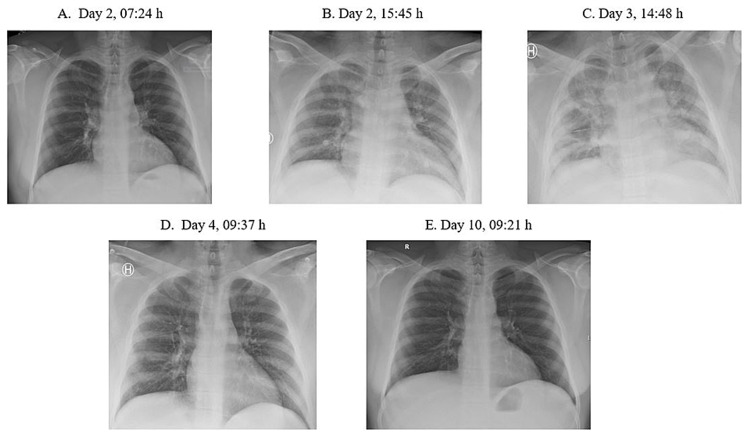
Images of chest x-rays showing the development (A-C) and subsequent regression of pulmonary infiltrates (D, E)

On suspicion of pneumonia and/or ACS, we administered oxygen supplementation and intravenous cefotaxime (2 g BID) and erythromycin tablets (500 mg BID) in addition to subcutaneous dalteparin (5000 IE OD), all according to institutional guidelines. Still, in the evening, he became increasingly somnolent and confused, and he was transferred to the intensive care unit. As shown in Table 1, Hb dropped and hemolysis increased as evidenced by increased concentrations of lactate dehydrogenase and bilirubin. Concomitantly, marked pulmonary infiltrates developed as shown in Figure 1. 

On vital indication, the next day (day 3), we performed a total exchange transfusion with 20 cross-matched units of packed red blood cells and six units of plasma (body weight: 110 kg). His clinical condition markedly improved thereafter, and he was transferred to the general ward on day 4. He was discharged on day 10 with oral antibiotics and painkillers. In the following four weeks, his condition continued to improve, both clinically and according to laboratory results (Table 1).

## Discussion

Although no universally accepted definition of ACS exists, most institutions and experts would require the presence of at least one of the following signs and symptoms for a diagnosis: fever, dyspnea, tachypnea, chest pain, and cough, and reduced blood oxygen saturation in addition to the development of pulmonary infiltrates [2,3]. During the initial two days in the ward, our patient fulfilled all these criteria, and thus, he clearly had acquired ACS.

Newer data are scarce regarding the prevalence of ACS. Castro et al. reported in 1994 that nearly 30% of 3751 studied SCD patients of all ages from the United States developed at least one episode of ACS [6]. The prevalence in Norway is unknown, but in our institution, we manage about 20 adult SCD patients of whom one to two patients are admitted to the hospital for ACS per year.

The etiology triggering ACS in SCD also remains under-investigated. In one of the largest reports of ACS to date, Vichinsky et al. [3] found in their US multicenter (n = 30) study conducted between 1993 and 1997 that among 538 SCD patients (all ages), pulmonary fat embolism and infectious microbes were the two dominant causes. Our patient had a normal CRP value, no abnormality on chest x-ray, and no fever upon hospital admittance. Later on, CRP increased markedly, and he developed both fever and bilateral pulmonary infiltrates (Table 1 and Figure 1). Thus, although no pathogenic microbe was detected, we cannot rule out that an infection was the triggering cause of his ACS. During the hospital stay, he received anticoagulation. So even though D-dimer increased (Table 1), pulmonary embolism is less likely to have occurred. Notably, the lung circulation may still have been affected, leading to a temporary cardiac failure as indicated by the elevated pro-BNP-level (Table 1).

Both Hagar et al. [7] and Allareddy et al. [8] found that HbSS was the dominant genotype (>80%) among SCD patients who developed ACS; however, compound heterozygotes (i.e., HbS-thalassemia and HbSC) were also diagnosed with ACS. Our patient had a particular genotype involving three different mutations (HbS, G6PD, and alpha-thalassemia), but whether this triple mutation increased the risk of ACS is unknown. The habitual Hb concentration in our patient was only moderately lowered (~9 g/dl), consistent with previous findings in patients with similar triplet mutations [9]. Reportedly, SCD patients who are heterozygote for HbS and alpha-thalassemia usually have mild hemolytic anemia [10,11]. In contrast, studies in SCD patients with concomitant G6PD have shown no clear effect regarding the degree of hemolytic anemia [12,13].

ACS is a leading cause of mortality among SCD patients and has been reported as high as 30% [3,8]. In their large US study comprising 24,699 hospitalizations due to ACS among adult SCD between 2004 and 2010, Allareddy et al. [8] identified the need for mechanical ventilation as an independent predictor for in-hospital mortality. There is no consensus as to what constitutes the optimal treatment for ACS, but several institutions advocate blood transfusion [8,14]. However, there is little evidence to support this and, in particular, if one should administer just simple transfusions of packed red blood cells (40%-50% of ACS cases) or perform more elaborate partial or total exchange transfusions therapy (3%-6% of ACS cases) [2]. Notably, there are no adequately powered randomized controlled trials or systematic reviews/meta-analyses to guide the treating physician [2]. Due to the rapid deterioration of our patient, we decided to administer a full blood exchange transfusion in addition to intravenous antibiotics and fluids, leading to a swift change in his clinical condition and improvement of biomarkers of inflammation, respiration, and lowering of HbS. Notably, mechanical ventilation was not considered necessary.

## Conclusions

While there is ample evidence concerning the efficacy of hydroxyurea for SCD patients, including prevention of ACS, it is unknown if switching from hydroxyurea to exchange transfusion is equally effective in preventing ACS. Our patient had only experienced ACS once before the current episode. He stopped hydroxyurea about six months before the current hospital admission due to his wish to be a father again. During that period, he received his partial exchange transfusions as long as seven to eight weeks apart, and his HbS fraction on hospital admission was about 55%, clearly above the desired level of 30%. Possibly, this transfusion regime was inadequate in protecting him from ACS. As such, this is a rare case report of such a trigger of ACS.
